# Reducing Diabetic Ketoacidosis in Pediatric Type 1 Diabetes: The Impact of Screening Programs and Early Disease-Modifying Treatment

**DOI:** 10.3390/jcm15155865

**Published:** 2026-07-27

**Authors:** Yung-Yi Lan, Rujith Kovinthapillai, Andrzej Kędzia, Elżbieta Niechciał

**Affiliations:** 1 Center for Medical Education in English, Poznan University of Medical Sciences, 41 Jackowskiego St., 60-512 Poznań, Poland; catherine974u@gmail.com (Y.-Y.L.); rujithk22@icloud.com (R.K.); 2 Department of Pediatric Diabetes, Auxology and Obesity, Poznan University of Medical Sciences, ul. Szpitalna 27/33, 60-572 Poznań, Poland; akedzia@ump.edu.pl

**Keywords:** diabetic ketoacidosis, type 1 diabetes, pediatric diabetes, screening programs, autoantibodies, immune modulation, disease-modifying therapies, DKA prevention

## Abstract

**Background:** Diabetic ketoacidosis (DKA) remains a preventable yet frequent complication at the onset of type 1 diabetes (T1D) in children, driven by delayed symptom recognition, socioeconomic disparities, and inconsistent access to care. Early identification of presymptomatic T1D through autoantibody-based screening, together with emerging disease-modifying therapies, may reduce the incidence of DKA. This review synthesizes evidence on epidemiology, risk determinants, screening strategies, and immunological interventions relevant to DKA prevention. **Methods:** A narrative review was conducted using PubMed, EMBASE, Scopus, Web of Science, and Google Scholar (2011–2026). Eligible sources included clinical studies, guidelines, systematic reviews, meta-analyses, and prevention trials addressing staging, screening, epidemiology, and disease-modifying treatments in pediatric T1D. Landmark publications outside this timeframe were included when essential. Evidence was integrated to identify determinants of DKA and strategies to reduce its occurrence. **Results:** DKA risk is influenced by younger age, socioeconomic disadvantage, rural residence, misdiagnosis, and limited access to specialized care. Sustained public awareness and community-based education reduce DKA incidence, whereas short-term campaigns show transient effects. Structured screening programs, including TrialNet and TEDDY, demonstrate near-elimination of DKA among monitored children. Teplizumab delayed progression from stage 2 to stage 3 T1D by a median of approximately 24 months in the original TN-10 trial, with extended follow-up demonstrating a median delay of 32.5 months. It is approved for individuals with stage 2 T1D aged ≥ 1 year and has recently gained approval for selected patients with newly diagnosed T1D, expanding opportunities for early disease modification. Global networks such as INNODIA strengthen prevention through coordinated biomarker-driven research. **Conclusions:** Reducing DKA at T1D onset requires integrated, sustained strategies combining public awareness, systematic autoantibody screening, structured follow-up, and access to emerging immunotherapies. Coordinated care across primary providers, pediatric endocrinologists, and research networks is essential to advance a proactive, prevention-oriented model of pediatric T1D care.

## 1. Introduction

Diabetic ketoacidosis (DKA) is a dangerous and potentially life-threatening complication that often accompanies the initial diagnosis of type 1 diabetes (T1D) in children. Despite being largely preventable, DKA continues to affect a substantial proportion of newly diagnosed pediatric patients, especially in settings where early recognition of T1D symptoms is delayed.

DKA frequently occurs as the first presentation of T1D, particularly in children under 5 years of age and in individuals facing barriers to healthcare. It remains a major clinical concern in pediatric diabetes [1,2]. Depending on social factors such as public awareness, socioeconomic status, access to medical care, and human development index (HDI), the percentage of DKA at the onset of T1D diagnosis varies greatly among populations, ranging from 15.6% to as high as 78.5%, with a pooled global prevalence being 41.9% [3]. It is generally believed that there is an inverse relationship between DKA frequency at T1D diagnosis and T1D incidence, perhaps due to earlier recognition of T1D symptoms and prompt diagnosis and interventions before progression to DKA [4].

Global prevalence of DKA at the onset of T1D varies significantly across regions and countries. Although efforts have been made to raise the awareness of T1D across medical professions and the general public, a substantial increase in the prevalence of DKA at T1D onset in children has been observed in Austria, Italy, Luxembourg, Slovenia, Poland, and the United States [5,6,7]. On the contrary, Australia, Denmark, Germany, Norway, Sweden, and Wales have reported significantly lower prevalence of DKA at T1D onset [5,8]. Data regarding the regional or national prevalence of DKA at T1D onset can have profound implications for the implementation of population-based screening programs for early-stage T1D, especially in children and adolescents at genetic or familial risk [9]. Furthermore, they may influence momentum for public education campaigns; guidance for targeted education for healthcare providers, educators, and parents; resource allocation; and support for regulatory and policy decision-making [8,9].

Recent epidemiological trends have also been affected by the coronavirus 2019 (COVID-19) pandemic. Meta-analyses and multinational cohort studies revealed a significant increase in both the incidence and severity of DKA at the onset of T1D during the pandemic compared with pre-pandemic periods, with relative risk estimates ranging from 1.41 to 1.44, and up to 1.66 for severe DKA [10,11]. While several mechanisms have been proposed to explain the association between severe acute respiratory syndrome coronavirus 2 (SARS-CoV-2) infection and β-cell dysfunction, the increased incidence of DKA has been attributed primarily to disruptions in healthcare delivery and access, stringent public health containment measures, delayed medical evaluation and diagnosis, and fear of exposure to SARS-CoV-2 [8]. These findings highlight the vulnerability of DKA prevention efforts and the importance of timely recognition of T1D symptoms, as well as uninterrupted access to pediatric diabetes services, even during public health emergencies.

Depending on the severity of DKA at the onset of T1D and whether early detection and intervention are achieved, DKA can have short- and long-term consequences for health outcomes. Severe metabolic derangements and dehydration in the acute phase can precipitate acute kidney injury (AKI), a common complication of DKA that may further exacerbate multi-organ injury involving the kidneys and brain [12]. Beyond immediate or short-term effects, a single event of moderate or severe DKA at T1D diagnosis in young children has been shown to negatively affect cognitive scores and alter brain growth [13]. In the long run, DKA at diagnosis predicts poorer glycemic control that is independent of demographic and socioeconomic factors, indicating a lasting impact on T1D management and disease trajectory [14,15]. These findings, collectively, highlight DKA as not only a life-threatening complication but also a key contributing factor for neurocognitive development, renal health, and long-term metabolic outcomes [16].

This article highlights the importance of early detection of T1D in children, with a particular focus on population-based and targeted screening strategies as key approaches to reducing the incidence of DKA at disease onset. In addition, the article discusses emerging therapeutic options that could modulate the course of pre-symptomatic T1D in children and delay its clinical manifestation.

## 2. Materials and Methods

This review used a narrative approach rather than a systematic literature review. This method enabled the incorporation of evidence from a wide range of sources, including clinical guidelines, real-world practice observations, and data from various study designs and clinical trials. This diverse perspective was necessary for comprehensively addressing the multifaceted epidemiological, clinical, preventive, and therapeutic aspects of DKA at T1D onset in children and adolescents.

A literature search was performed between October 2025 and May 2026 across PubMed, EMBASE, Scopus, Web of Science, and Google Scholar. Search terms were selected based on key concepts, including DKA, T1D, pediatric populations, screening, staging, prevention, and disease-modifying therapies. Representative search strings and Boolean operators included (“diabetic ketoacidosis” OR “DKA”) AND (“type 1 diabetes” OR “T1D” OR “autoimmune”) AND (“child*” OR “pediatric” OR “adolescent*”) AND (“new-onset” OR “newly diagnosed”) AND (“prevalence” OR “incidence” OR “frequency”) AND (“screening” OR “early detection” OR “prevention”) AND (“treatment*” OR “disease-modifying” OR “immunotherapies”). Additional database-specific searches incorporated terms related to epidemiology, risk factors, healthcare access, and therapeutic interventions.

Articles published between 2011 and 2026 were screened for clinical relevance by title, abstract, and full text. However, landmark publications outside this time range were included when considered essential to the objectives of the review and the historical or conceptual context. Inclusion criteria comprised clinical studies, narrative reviews, systematic reviews, meta-analyses, and international guidelines focused on the epidemiology, risk factors, staging, screening strategies, prevention, and disease-modifying treatment of T1D, particularly regarding reducing DKA at diagnosis. Exclusion criteria comprised studies with insufficient data or inaccessible full texts, non-English publications, non-peer-reviewed literature, duplicate studies, and abstract-only texts.

The literature search identified 466 records; after screening, 379 publications were evaluated, and 145 were included in the final narrative review. Study selection and interpretation were reviewed independently by the authors involved in manuscript preparation. Discrepancies in interpretation or selection were resolved through discussion until consensus was achieved among the authors. Due to the narrative nature of the review, no formal risk-of-bias assessment and quality appraisal tools were applied. Priority was given to higher levels of evidence, such as international guidelines, randomized controlled trials, original research, large observational studies, and systematic reviews, to ensure robust clinical applicability. The selected literature offers both theoretical insights and practical frameworks pertinent to reducing DKA at T1D onset in the pediatric population.

The authors used generative artificial intelligence (GenAI) to create the graphical abstract and figure included in this manuscript.

## 3. Risk Factors for DKA at T1D Onset

The risk factors for DKA at the onset of T1D in children are multifactorial, involving demographic, socioeconomic, diagnostic, and healthcare-related determinants. Social inequalities contribute to a major disparity in presentation, where children from low-income households and those lacking insurance are especially vulnerable to delayed diagnosis and are more likely to present with DKA upon T1D onset [17,18,19,20]. Geographically, children living in rural areas often experience delayed diagnosis because of limited healthcare infrastructure and inadequate access to specialized care, as early access or referral to pediatric endocrinology services is frequently unavailable or postponed [18,19,21]. Furthermore, younger age at onset, ethnic minorities, and the absence of a family history of diabetes are all associated with an increased likelihood of DKA at T1D onset, largely due to difficulties in recognizing early signs of hyperglycemia [5,17,20,22,23].

While socioeconomic disparities play a central role, healthcare-related factors, such as misdiagnosis or delayed diagnosis, can contribute to DKA at T1D onset. Early signs of diabetes, including polyuria, polydipsia, unexplained weight loss, fatigue, and blurry vision, may be misattributed to common pediatric illnesses such as viral infections, urinary tract infections, or gastroenteritis [17,22]. These conditions are not only leading causes of diagnostic confusion but may also act as physiological stressors that precipitate DKA [24]. This delay in diagnosis can result in progressive metabolic decompensation, culminating in DKA and potentially coma and death [25]. Thus, the ability for primary care and emergency physicians to recognize early warning signs is imperative for providing prompt interventions. Increased awareness and simple diagnostic tools, such as routine blood glucose or urine ketone testing, in symptomatic children can markedly reduce DKA rates at T1D diagnosis [9,17,26].

The COVID-19 pandemic provided a real-world example of how disruptions in healthcare access and diagnostic pathways can increase DKA risk at T1D diagnosis. Lockdowns, disrupted access to healthcare services, delayed medical consultations and diagnosis, and parental hesitancy to seek medical attention because of fear of exposure to the virus resulted in increased DKA rates and severity in new-onset T1D patients [8]. As a result, many children experienced advanced metabolic decompensation upon presenting to hospitals. This phenomenon was supported by registry analyses and multicenter studies from Germany, the UK, and the US, which reported up to a twofold increase in DKA incidence at T1D onset during 2020–2021. The increase was particularly pronounced among younger children (<6 years) and those from socioeconomically disadvantaged populations, underscoring the multifactorial and overwhelming impact of the pandemic on DKA and T1D detection, care, and outcomes [11,27,28].

On a systemic level, the healthcare system infrastructure and existing health policies can strongly affect early detection and DKA prevention. Countries equipped with structured public health programs, such as diabetes awareness initiatives in schools and communities, national screening programs targeted to identify individuals at risk for T1D, or well-organized diabetes care pathways, report substantially lower rates of DKA at T1D diagnosis. This association is supported by findings from The Environmental Determinants of Diabetes in the Young (TEDDY) and Trial to Investigate Glucose Tolerance (TRIGR) studies [8,17,29,30]. Data from a meta-analysis and an awareness campaign report have shown that community-based screening and targeted education campaigns could reduce DKA incidence by 7–12% [7,31]. Yet other systematic reviews found no statistically significant overall benefit. Regardless, systems with absent coordinated pediatric diabetes education and training or standardized diagnostic protocols frequently encounter a higher prevalence of DKA at diagnosis [18,26]. Therefore, comprehensive policy measures that promote clinician education and training, improve healthcare accessibility, and raise public awareness of T1D and its early warning signs are essential for mitigating structural risk factors for DKA at T1D onset in children.

Despite evidence supporting the effectiveness of community-based screening and targeted education in reducing the frequency of DKA at T1D diagnosis by approximately 7%, the long-term sustainability of these awareness campaigns remains a significant challenge [7,17]. Previously reported initiatives have produced mixed long-term results, primarily because measurable reductions in DKA rates are often confined to the active campaign period. Once these programs conclude, the incidence of DKA frequently reverts to pre-campaign levels. This transient effect was notably demonstrated in a Canadian multi-intervention project, in which pediatric DKA hospitalization rates decreased during a 6-year active campaign but returned to baseline shortly after the project ended [6]. Conversely, continuously maintained campaigns demonstrate sustained efficacy. For example, the extensively studied Parma campaign in Italy dramatically reduced DKA prevalence at diagnosis from 78% to 12.5%; because the intervention was renewed and maintained, its protective effects persisted for years and even spontaneously disseminated to neighboring provinces [32]. The Stuttgart campaign in Germany further corroborated this, emphasizing that awareness programs are most successful when consistently maintained [31,33]. Consequently, while public awareness campaigns are effective acute interventions, their potential to diminish upon cessation underscores the necessity for continuous, rather than episodic, public health efforts. The permanent integration of structured diabetes education into standard pediatric care and community health frameworks is essential to maintaining lower DKA rates over time [34,35,36]. Despite these encouraging findings, most evidence supporting screening programs is derived from prospective cohort studies rather than randomized trials. Although studies such as TEDDY and TrialNet consistently demonstrate markedly reduced DKA rates among monitored children, participants received intensive follow-up within specialized research settings, which may limit the generalizability of these findings to routine clinical practice [21,23,37]. Further implementation studies are needed to evaluate the effectiveness and cost-effectiveness of population-based screening programs across diverse healthcare systems.

## 4. Staging of T1D and Its Implications

Our understanding of T1D has evolved over the past decade, from regarding it solely as an acute onset disease to recognizing it as a progressive autoimmune process that develops through identifiable stages [38,39]. The fundamentals of each stage have profound implications for clinical care, research, screening programs, treatment options, and regulatory settings. According to the International Society for Pediatric and Adolescent Diabetes (ISPAD) Clinical Practice Consensus Guidelines 2024, T1D is characterized by three established stages according to specific islet autoantibody (AAB) status, metabolic abnormalities, and clinical features [40]. Although some researchers and clinicians include an additional “Stage 4” to describe the period of established disease requiring long-term management, this terminology is not formally recognized in the current Juvenile Diabetes Research Foundation (JDRF), Endocrine Society, or American Diabetes Association (ADA) staging system, and remains non-standard [38].

- Stage 1 patients have normal glycemic levels and are presymptomatic. The diagnosis is made using validated assays that identify multiple islet AABs in at least two samples [9,39]. Prompt interventions are necessary because 44% of stage 1 children will progress to stage 3 T1D in 5 years, and 80 to >90% will progress within 15 years [9,38,39].
- Stage 2 patients, in addition to multiple islet AABs confirmed on at least two samples, have elevated fasting glucose or impaired glucose tolerance (IGT), detected through an oral glucose tolerance test (OGTT); elevated glycosylated hemoglobin (HbA1c) of 5.7 to 6.5% (39 to 48 mmol/mol); or a ≥10% change in HbA1c [9,41]. Depending on the extent of dysglycemia, patients can be further subclassified as stage 2a or stage 2b [39]. With elevated glycemic levels, 75% of stage 2 children will progress to stage 3 in 5 years and nearly 100% in their lifetime [9].
- Stage 3 involves hyperglycemia that meets the glycemic and clinical diagnostic criteria set by the ADA [9,41]. These patients may be either asymptomatic (stage 3a) or symptomatic (stage 3b), with the latter presenting with classic polyuria, polydipsia, and unexplained weight loss, requiring immediate insulin intervention [39,42].
- Stage 4 patients are referred to as having long-standing T1D, with individuals already requiring insulin therapy. While the standard staging framework established by the 2015 JDRF, Endocrine Society, and ADA scientific statement primarily defines three preclinical stages, the concept of a fourth stage captures the long-term clinical management and surveillance of disease progression through individualized therapy, complication prevention, and incorporation of therapeutic innovation, reflecting an evolving classification system [38,40,43].

**Figure 1:**
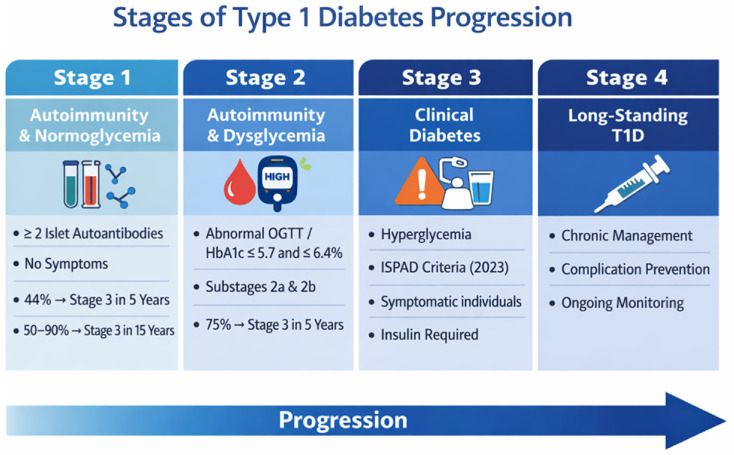
An overview of the four stages of type 1 diabetes, highlighting key diagnostic features and risk of progression. (Abbreviations: T1D—type 1 diabetes; OGTT—oral glucose tolerance test; ISPAD—International Society for Pediatric and Adolescent Diabetes) [9,38,39,41,42,43].

Figure 1 summarizes the stages of T1D progression [9,38,39,41,42,43].

Ultimately, the identification of these distinct stages means that clear, evidence-based recommendations for monitoring children with positive islet autoantibodies are already available. The current ISPAD guidelines, international clinical care advice documents, and national Polish recommendations outline highly structured follow-up protocols for individuals identified through early screening programs [39,40,44]. In practice, implementing these recommendations requires a proactive clinical approach. Regular follow-up visits must be scheduled to assess key parameters of glucose metabolism, specifically including periodic evaluation of fasting glucose, continuous HbA1c monitoring, and oral glucose tolerance testing, alongside repeated measurements of autoantibody titers to track immunological activity [9,39].

During these scheduled assessments, clinicians must vigilantly monitor specific clinical and biochemical markers that indicate progression toward symptomatic disease. A progressive rise in HbA1c, worsening glucose tolerance during an OGTT, or the subtle onset of clinical symptoms such as mild polyuria or weight loss serve as critical red flags [39]. Early identification of these at-risk children enables intensified clinical monitoring and timely therapeutic intervention at the exact moment when metabolic deterioration begins [38,45].

Beyond strict monitoring, early staging offers an opportunity to explore targeted interventions to preserve β-cell function. Recent reports suggest that lifestyle modification, such as physical activity, dietary patterns, and weight management, may actively slow the progression of presymptomatic T1D in some individuals. By decreasing insulin resistance, weight management reduces the metabolic demand placed on the remaining functional β-cells [46,47]. Observational data from the TEDDY study found that moderate to vigorous physical activity was associated with a reduced risk of progression to T1D in children with multiple islet autoantibodies (hazard ratio (HR) 0.92 per 10 min increase; 95% confidence interval (CI) 0.86–0.99), and higher glycemic index food consumption was associated with an increased progression risk (HR 2.20; 95% CI 1.17–4.15) [47]. Additionally, data from TEDDY, TrialNet, and other longitudinal cohorts link elevated BMI and adiposity to accelerated progression through preclinical stages, with obesity improving the performance of progression risk stratification models [46,48]. One observational report noted that significant weight loss (approximately 10% compared to baseline) may prevent or delay T1D onset in around 20% of cases [49]. However, these findings remain preliminary and require confirmation in prospective interventional studies.

Overall, current evidence supporting lifestyle modification is based primarily on observational studies, and no randomized interventional trials have demonstrated that lifestyle interventions targeting physical activity, dietary patterns, or weight management can prevent progression from stage 1 or stage 2 T1D [47]. Consequently, lifestyle interventions cannot currently be considered established disease-modifying therapies. Nevertheless, in the current era of rapidly developing disease-modifying therapies, most notably teplizumab, which delayed progression from stage 2 to stage 3 T1D by a median of approximately 24 months in the original TN-10 trial, with extended follow-up demonstrating a median delay of 32.5 months, patients who decline or are not eligible for this therapy may still benefit from structured lifestyle interventions combined with close clinical surveillance [50,51]. Ultimately, increased awareness paired with regular, protocol-driven monitoring substantially reduces the risk of DKA at the time of clinical diagnosis, as demonstrated by screening programs where DKA prevalence was reduced to less than 5% compared to over 20 to 40% in unscreened populations [37,38,45].

## 5. Strategies to Reduce DKA Incidence at T1D Onset

Measures to reduce DKA incidence at T1D onset require a combination of public health campaigns, policy implementations, educational initiatives, physician continuing education, and targeted screening programs. Awareness campaigns aimed at parents, teachers, school staff, healthcare professionals, and even pupils can significantly decrease the frequency of DKA at T1D diagnosis by promoting recognition of early T1D symptoms. For instance, the 4Ts (toilet, tired, thinner, and thirst) campaign launched by Diabetes UK utilized national press, television ads, radio ads, social media platforms, parent and child magazines, and posters placed in doctors’ offices to reach a wide audience, particularly parents [52,53]. Table 1 summarizes major screening programs and their impact on DKA at the time of T1D diagnosis.

Community-based education programs play an equally vital role in bridging the gap between symptom onset and clinical presentation. The Stuttgart Diabetes Awareness Campaign, conducted from 2015 to 2017, significantly reduced the incidence of DKA at T1D onset in children and adolescents from 28% to 16%, with the most notable effect observed in children under six years old [31]. By hosting regular public activities and distributing informational flyers and posters at school entry health examinations, in day-care centers, and in all pediatric practices, the campaign targeted not only families and teachers but also general practitioners, pediatricians, and public health staff [31]. Similarly, regional awareness campaigns, such as those in Parma, have shown marked reductions in DKA incidence through distribution of posters in schools, training for pediatricians and healthcare providers, and toll-free information lines [63]. The sustained reduction in DKA rates can be attributed to improved recognition of early symptoms and close cooperation between the children’s hospital and the public health department, which enables prompt referral and early intervention [31,33].

Italy’s nationwide screening law exemplifies large-scale efforts to implement early detection strategies, the Italian Republic Law 130/2023, for T1D and celiac disease in children aged 1–17 years, initiated in 2023 with pilot programs in regions such as Lombardy, Marche, Campania, and Sardinia [57,58]. Coordinated by primary care pediatricians, screening uses capillary blood autoantibody testing to identify children in the presymptomatic stage of T1D [57]. Those who test positive for islet autoantibodies are enrolled in regional registries, through which they receive regular metabolic monitoring, symptom education, and coordinated oversight by family pediatricians and specialized diabetes centers, with rapid evaluation and swift referral if progression occurs [59].

Building on regional initiatives, participation in international programs such as TrialNet in Milan demonstrates that regular autoantibody screening and structured follow-up of at-risk relatives can effectively prevent DKA at diagnosis. TrialNet identifies first- and second-degree relatives of individuals with T1D through periodic testing for islet autoantibodies, including glutamic acid decarboxylase autoantibodies (GADAs), insulin autoantibodies (IAAs), insulinoma-associated antigen-2 autoantibodies (IA-2As), islet cell autoantibodies (ICAs), and zinc transporter 8 autoantibodies (ZnT8As) [37,56]. In the Milan cohort, none of the screened and followed relatives developed DKA at onset, indicating the effectiveness of structured surveillance and coordinated monitoring [37]. This prevention-oriented approach aligns with both ISPAD and ADA guidelines, promoting early detection and lowering the risk of DKA at diagnosis [9].

Beyond campaigns and education, nationwide screening initiatives, including T1D autoantibodies testing and/or genetic risk assessment, targeting at-risk pediatric populations, offer a more proactive approach to DKA prevention and early detection of T1D. The TEDDY study, which was launched in 2004, followed 8676 children with T1D-risk HKA-DR-DQ genotypes from the USA, Finland, Germany, and Sweden [54]. This multinational study demonstrated positive outcomes of screening programs, where none of the TEDDY participants developed DKA at diagnosis [55].

Collectively, evidence shows that combining awareness campaigns, educational efforts, and targeted screening programs can substantially reduce the risk of DKA at diagnosis. Continued investment in these complementary approaches and broader scaling of these efforts present a clear path toward improving early detection and the trajectory of newly diagnosed T1D. Although these findings are encouraging, much of the evidence supporting screening programs is derived from observational studies and structured research cohorts rather than randomized trials. Additional implementation and cost effectiveness studies are required to determine whether similar benefits can be achieved in routine clinical practice.

## 6. Advances in T1D Prevention and Disease Modulation

Over the past decade, notable progress has been made in the early detection, prevention, and immunological modulation of T1D, an autoimmune disorder marked by T-cell-mediated destruction of pancreatic β-cells. Although the disease often begins in childhood and insulin remains essential, emerging disease-modifying therapies are reshaping management.

Advances in understanding of T1D pathogenesis and the availability of biomarkers, particularly islet autoantibodies, now support the identification of presymptomatic stages and recruitment into prevention trials. Screening is recommended for genetically at-risk individuals, and major international societies underscore the value of early detection and intervention [56,64,65,66,67].

A landmark development is teplizumab, the first U.S. Food and Drug Administration (FDA)-approved therapy to delay progression from stage 2 to stage 3 T1D in at-risk individuals aged ≥8 years. By inducing partial CD3/TCR signaling and promoting exhaustion of autoreactive CD8+ T cells alongside expansion of regulatory T cells (Tregs), teplizumab helps restore immune tolerance and delayed progression from stage 2 to stage 3 T1D by a median of approximately 24 months in the original TN 10 trial, with extended follow up demonstrating a median delay of 32.5 months [50,51,68]. Other immunotherapies, such as abatacept, rituximab, anti-thymocyte globulin, and golimumab, demonstrate temporary preservation of β-cell function but lack durable effects and are not approved for prevention. Trials of oral insulin and probiotic strategies in genetically susceptible children are ongoing, though efficacy is not yet established [66,69]. Table 2 presents an overview of pharmacologic options for the prevention and modulation of disease in T1D.

Global initiatives such as TrialNet and INNODIA have advanced biomarker discovery and prevention-focused research. Current efforts emphasize combination therapies, personalized immunomodulation, and novel pathway-targeted agents, which leading organizations identify as key future directions in T1D prevention [66,69,88].

### 6.1. Teplizumab

Teplizumab (Tzield) represents a major advance in delaying diabetes (T1D) progression, as it is the first disease-modifying therapy capable of delaying the onset of clinical type T1D in individuals with early-stage disease [50,89]. Initially gaining FDA approval for individuals aged ≥8 years with stage 2 disease based on the pivotal TN-10 trial, this critical milestone has recently been expanded, with current FDA labeling now indicating approval for patients aged ≥1 year [50,70,90]. Furthermore, its availability is expanding globally; teplizumab is now approved in the UK by the Medicines and Healthcare products Regulatory Agency (MHRA), as well as in Israel and Saudi Arabia, and is available through compassionate use programs in several European nations, including France, Germany, Italy, and Spain [64,91]. This international availability marks an important shift toward presymptomatic immunological intervention rather than reliance solely on symptomatic management [68,71].

Teplizumab is a humanized anti-CD3 monoclonal antibody targeting the CD3ε chain on T cells [68,90]. Through partial agonistic signaling of the CD3/TCR complex, it induces transient activation followed by functional exhaustion and anergy of autoreactive CD8+ and CD4+ T cells, while promoting expansion of regulatory T cells [64,68,92]. These effects help reestablish immune tolerance and slow β-cell destruction. Teplizumab also downregulates interleukin-7 (IL-7) receptor expression on CD8+ T cells, limiting their proliferation and pathogenic expansion [64,92].

Teplizumab delayed progression from stage 2 to stage 3 T1D by a median of approximately 24 months in the original TN-10 trial, with extended follow up demonstrating a median delay of 32.5 months [47,50,51,93]. The most common adverse events, transient lymphopenia and rash, were typically mild and self-limiting [50,70]. Teplizumab currently has the strongest clinical evidence among disease-modifying therapies for T1D because its efficacy has been demonstrated in randomized placebo-controlled trials. Nevertheless, additional long-term data are needed to better define its durability, accessibility, and implementation in routine clinical practice [47,93].

Teplizumab’s mechanism and clinical impact have strengthened the staged model of T1D, supporting targeted intervention during the presymptomatic phase and establishing immune modulation as a viable strategy for disease prevention and delay [51,68]. With its expanding international regulatory approvals alongside its established presence in the United States, professional organizations such as the ADA now strongly recommend discussing teplizumab with eligible individuals in specialized clinical settings, ensuring that a broader global population can benefit from this groundbreaking therapy [47].

### 6.2. Regulatory T-Cell Therapy: Engineering Immune Tolerance

Regulatory T-cell (Treg) therapy is a leading investigational strategy for restoring immune tolerance in T1D [94,95]. Tregs, defined by the CD4^+^CD25^hi^CD127^lo/−^FOXP3^+^ phenotype, are critical for suppressing autoreactive immune responses and maintaining β-cell integrity [96,97]. Deficiency or dysfunction of Tregs is a hallmark of T1D pathogenesis, and genetic susceptibility loci for T1D are closely linked to Treg development and function [94,98,99].

Clinical trials of adoptive Treg transfer have consistently demonstrated safety but limited efficacy. In a recent phase 2 randomized trial, single infusions of autologous polyclonal expanded Tregs in children with new-onset T1D were well tolerated, with transient increases in activated memory Tregs, but did not prevent C-peptide decline over one year compared to placebo [72]. Similar safety profiles and modest efficacy signals have been reported in adults, with some individuals showing persistent C-peptide levels up to two years post-infusion [97,100]. Efficacy appears greatest in patients with shorter disease duration and higher residual β-cell mass, suggesting that early intervention may be critical for clinical benefit [101,102].

Barriers to efficacy include lack of antigen specificity, limited in vivo persistence, and instability of the Treg phenotype in inflammatory environments. Polyclonal Tregs may not efficiently target islet-specific autoimmunity, and their suppressive function can be compromised by the cytokine milieu of T1D. Technical challenges in ex vivo expansion and variability in Treg quality further complicate clinical translation [95,103,104,105].

Next-generation strategies focus on engineering Tregs for enhanced specificity and stability. Antigen-specific Tregs, generated by transducing T cells with engineered T cell receptors (TCRs) or chimeric antigen receptors (CARs), show improved targeting of pancreatic islet antigens and greater suppressive capacity in preclinical models [103,105]. For example, GNTI-122, an autologous engineered Treg therapy, demonstrated stable FOXP3 expression, targeted trafficking to the pancreas, and robust suppression of islet-specific effector T cells in vitro and in vivo [106]. Molecular approaches to reinforce Treg stability—such as optimizing cytokine signaling (IL-2, IL-33/ST2, IL-35), managing FOXP3 splicing, and leveraging epigenetic mechanisms—are under active investigation to address Treg plasticity and enhance durability [107,108,109].

Regulatory cell therapy may be most effective when combined with other immunomodulatory agents or β-cell protective strategies, such as low-dose IL-2, checkpoint inhibitors, or agents that reduce β-cell stress [101,110]. Myeloid-derived suppressor cells (MDSCs) and mesenchymal stem cells (MSCs) represent additional regulatory cell types under study for synergistic immune tolerance induction [101,111]. Artificial intelligence-driven patient stratification and personalized timing of intervention are emerging tools to optimize trial design and clinical outcomes [110,112].

### 6.3. Oral Insulin Therapy

Oral insulin therapy as an antigen-specific approach for T1D prevention has shown safety and immunomodulatory effects, but its efficacy is limited to genetically and immunologically defined subgroups, and combination strategies may enhance its tolerogenic potential [64,73,74].

Mechanism and Gut-associated Lymphoid Tissue (GALT) Effects: Oral insulin is designed to induce immune tolerance by presenting insulin peptides to the GALT, promoting regulatory immune responses rather than pathogenic autoimmunity [75,113,114]. This route is considered more tolerogenic than parenteral administration, based on preclinical and early human studies [113,115].

The Diabetes Prevention Trial of Type 1 (DPT-1) and TrialNet Outcomes: The DPT-1 and subsequent TrialNet studies found that oral insulin (7.5 mg/day) did not prevent diabetes in the overall population of autoantibody-positive relatives [64,74,76]. However, post hoc analyses revealed that individuals with high insulin autoantibody titers or impaired first-phase insulin release (FPIR) experienced a delay in progression to clinical diabetes, suggesting benefit in select subgroups [64,71,74]. Recent reanalysis of TrialNet data (TN07) showed that oral insulin delayed progression to stage 3 T1D in participants with the HLA DR4-DQ8 haplotype and/or elevated IA-2A (HR 0.59, *p* = 0.027), with the greatest effect in those with both risk markers [64,77].

Warsaw Oral Insulin Study and the Primary Oral Insulin Trial (POInT): The Warsaw Oral Insulin Study and the POInT evaluated high-dose oral insulin (up to 67.5 mg/day) in genetically at-risk, pre-symptomatic children [73,75,78]. Overall, oral insulin did not prevent the development of islet autoantibodies or diabetes in the general cohort (HR 1.12, *p* = 0.57), but a prespecified subgroup with the susceptible INS genotype showed reduced progression to diabetes or dysglycaemia (HR 0.38, 95% CI 0.17–0.86) [73,78]. These findings highlight the importance of genetic stratification for future antigen-specific prevention trials.

Combination Strategies: Combining oral insulin with adjuvants or immunomodulators such as low-dose IL-2 or teplizumab may enhance immune tolerance by promoting Treg expansion and modulating the immune environment [64,69,110]. Preclinical and early clinical data suggest that such combinations could condition the immune system for more robust and durable tolerance, but definitive efficacy data in T1D prevention are still pending [69,110,115].

### 6.4. Emerging Immunotherapies

Emerging immunotherapies for T1D currently under clinical investigation include low-dose IL-2 to selectively expand Tregs, anti-interleukin-21 (anti-IL-21) monoclonal antibodies, Janus kinase (JAK) inhibitors to modulate inflammatory cytokine signaling, and verapamil as a β-cell protective agent [20,64,82,110,116,117,118]. Combination strategies that integrate immune modulation with metabolic protection are increasingly favored and are being evaluated in phase 2 and phase 3 clinical trials [20,64,119].

Low-dose IL-2 is being studied for its ability to preferentially expand and activate Tregs, thereby restoring immune tolerance without broad immunosuppression [79,120,121,122]. Early-phase trials have demonstrated safety and Treg expansion, and ongoing studies are assessing efficacy in preserving β-cell function and delaying disease progression [80,81,83,123].

Anti-IL-21 monoclonal antibodies and JAK inhibitors (such as baricitinib, abrocitinib, and ritlecitinib) target key cytokine pathways involved in islet inflammation and autoimmunity [64,82,83,84]. JAK inhibitors offer the advantage of oral administration and have shown promise in preserving residual β-cell function and improving glycemic control in recent-onset T1D, with ongoing pivotal trials evaluating their long-term efficacy and safety [64,82,124].

Verapamil, a calcium channel blocker, reduces β-cell endoplasmic reticulum (ER) stress and apoptosis by downregulating thioredoxin-interacting protein [85,125,126]. Randomized clinical trials in newly diagnosed pediatric T1D have shown that verapamil can modestly preserve C-peptide secretion, and combination regimens with immunotherapies are under active investigation [85,86,127].

Current consensus supports multi-targeted interventions that combine immune modulation (e.g., teplizumab, abatacept, golimumab, anti-thymocyte globulin (ATG), JAK inhibitors) with agents that protect or restore β-cell health (e.g., verapamil, glucagon-like peptide-1 (GLP-1) agonists). These combination strategies are being evaluated in phase 2 and 3 trials, with the goal of achieving more durable disease modification and functional β-cell preservation than monotherapy alone [20,64,87,119,128]. Strategic sequencing or concurrent administration of immune and metabolic therapies is expected to enhance both the magnitude and durability of therapeutic effects [64,119,129,130]. The strength of evidence varies considerably among these therapies. While teplizumab is supported by randomized placebo-controlled trials and regulatory approval, most other immunotherapies remain investigational, with evidence limited to early-phase clinical trials or observational studies. Larger randomized studies with longer follow-up are required before these therapies can be incorporated into routine clinical practice [47,50,64].

### 6.5. Global Research Networks and Collaborative Prevention Initiatives

In recent years, global research efforts focused on T1D prevention have grown rapidly, driven by advances in our understanding of the underlying autoimmune mechanisms and the emergence of increasingly sophisticated biomarker technologies for monitoring disease progression and therapeutic responsiveness [131,132]. The INNODIA consortium, the largest European-based public–private network, exemplifies one of the most comprehensive attempts to integrate clinical research, multidisciplinary partnership, mechanistic studies, and longitudinal monitoring of people living with T1D. INNODIA DETECT aims to improve early detection of T1D in the general population by focusing on implementing and assessing strategies for population-level screening for islet autoantibodies [60]. Building on the foundational infrastructure of INNODIA, INNODIA HARVEST utilizes coordinated biobanks, standardized data collection, and streamlined multicenter, multinational clinical trials to test novel immunomodulatory or disease-modifying agents in at-risk individuals, with the goal to refine risk stratification and transform mechanistic insights into practical interventions [133]. Across Europe and around the world, parallel initiatives help reinforce this framework, fostering large collaborative studies that produce high-quality datasets necessary to accelerate research advances [134].

## 7. Discussion and Future Perspectives

Despite substantial progress in understanding the pathophysiology and predicting T1D in children and adolescents, there remains an urgent need to establish standardized, evidence-based screening guidelines for individuals and populations at elevated risk. The ADA recommends autoantibody-based screening for presymptomatic T1D children with a family history or known elevated genetic risk [9]. However, current screening practices, ranging from autoantibody testing in first- and second-degree relatives of individuals with T1D to population-based pilot programs, vary greatly across regions and countries, reflecting discrepancies in health care system capacity, availability of laboratory infrastructure, and financial constraints [9,20,135,136]. While screening has the potential to reduce DKA incidence and facilitate timely intervention, large-scale implementation requires careful evaluation of cost-effectiveness alongside practical considerations such as access to screening, disparities between high- and low-resource healthcare systems, ethical and psychosocial issues, screening frequency, target populations, follow-up requirements, and downstream healthcare resource utilization [135,137,138]. Expanding screening beyond high-risk groups raises ethical and practical concerns, such as how to handle informed consent, the potential psychological burden on the patient and family, and the challenges of identifying children at risk when preventive measures are not yet fully established [20,136]. Overall, the available evidence supports early identification of individuals at risk for T1D as an effective strategy to reduce DKA at diagnosis. However, the certainty of evidence differs across preventive approaches, with randomized clinical trial data supporting teplizumab, whereas evidence for public awareness campaigns, screening implementation, and several emerging immunotherapies relies primarily on observational studies or early-phase clinical trials. Future multicenter implementation studies and randomized trials are needed to strengthen the evidence base and inform broader adoption of these strategies. As immunotherapies such as teplizumab gain wider recognition and regulatory approval, ongoing research must address their long-term efficacy, safety profile, durability of immune modulation, and cost-effectiveness. Beyond clinical efficacy, successful implementation will require infrastructure to identify eligible individuals, availability of teplizumab, consideration of treatment accessibility and delivery, infusion capacity, reimbursement policies, and the feasibility of long-term monitoring strategies [68,139]. These challenges are especially relevant in pediatric populations, where access to care, socioeconomic circumstances, family preferences, and adherence may influence clinical outcomes, treatment uptake, and the overall effectiveness of preventive care [140,141].

With continued progress in global research, there is a growing emphasis on embedding preventive strategies into routine clinical care [64,131]. Recommended actions include establishing standardized autoantibody screening for genetically at-risk children, incorporating validated risk stratification tools in primary and pediatric care, and instituting defined follow-up pathways for children identified early in the disease process [9,29,60,64,142]. As preventive immunotherapies achieve wider clinical validation, successful implementation will require clinicians to be competent at risk communication, equitable access to diagnostic and therapeutic tools, and coordinated care between primary care, endocrinology specialists, and research teams [20,29,60,64,143]. Altogether, these measures form a critical link through which scientific advances are transformed into actionable, prevention-focused clinical practice.

The COVID-19 pandemic further exposed the vulnerability of DKA prevention strategies due to disruptions in healthcare delivery. The rise in DKA incidence and severity highlighted that effective prevention through advances in screening and disease-modifying therapies also relies on resilient healthcare systems capable of maintaining timely diagnosis and continuity of diabetes care during public health crises [8,10]. Future prevention strategies and disease management should build on these lessons by integrating emergency preparedness into existing screening and care pathways, thereby minimizing diagnostic delays [144,145].

Future efforts to curb DKA at the time of T1D diagnosis will rely on integrated strategies that combine public awareness campaigns, early diagnosis initiatives, healthcare systems strengthening, and equitable implementation of preventive measures. Targeted screening programs and interventions are especially needed in resource-limited regions, where tailored strategies such as strengthening primary care capacity, enhancing community awareness, empowering first-contact health professionals, and broadening the availability of diagnostic tools are essential [17,39]. The path towards personalized medicine, driven by advances in genomics, autoantibody profiling, and metabolic staging, could enable individualized risk assessment and adapted prevention strategies. With the development of precision medicine, it may be possible to integrate biological, environmental, and digital biomarkers to redefine the landscape of T1D prevention and treatment, leading to reductions in DKA rates and promoting timely, individualized care [9,36,39]. Nonetheless, translating these advances into meaningful reductions in DKA will require context-specific implementation strategies that address disparities in infrastructure, healthcare access, and socioeconomic conditions across diverse populations.

## 8. Conclusions

DKA at the onset of T1D remains a prevalent yet largely preventable complication, influenced by socioeconomic inequalities, missed or delayed recognition of early symptoms, and variable access to timely medical care. Evidence from epidemiologic analyses, COVID-19-related data, and screening cohorts suggests that improving early detection and implementing structured care pathways can significantly reduce DKA at diagnosis. Current findings point to the need for multi-level interventions that integrate continuous public awareness initiatives and systematic AAB screening with structured follow-up, while leveraging the emerging capacity of novel immunotherapies to modulate the disease course. Successful adoption of these strategies requires strong coordination among primary care providers, emergency care physicians, pediatric endocrinologists, research networks, and public health authorities, enabling the translation of scientific findings into measurable clinical outcomes and improved patient care.

Although substantial progress has been made in early T1D detection and prevention, key knowledge gaps remain. Future work must optimize screening strategies, establish long-term effectiveness and cost-effectiveness of screening programs, evaluate the durability and efficacy of disease-modifying therapies, and identify implementation strategies to facilitate the integration of research into routine healthcare. Moreover, it is important to ensure equitable access to screening, specialist follow-up, and preventive therapies across diverse healthcare settings. Strengthening these efforts will be essential for decreasing global DKA rates and establishing a more proactive, prevention-oriented paradigm for T1D care.

## Figures and Tables

**Table 1:** A summary of major type 1 diabetes screening programs worldwide and their impact on diabetic ketoacidosis outcomes.

Program	Country/Region	Screening Method	Population	Follow-Up Strategy	DKA Outcome	Ref.
**TEDDY**	USA, Finland, Germany, and Sweden	Genetic risk screening and serial AAB monitoring	8676 high-risk children	Longitudinal metabolic surveillance	No participants developed DKA at diagnosis	[54,55]
**TrialNet**	International	Islet AAB screening	First- and second-degree relatives of individuals with T1D	Structured follow-up and metabolic monitoring	Marked reduction in DKA rates; Milan cohort reported no DKA cases at diagnosis	[37,56]
**D1Ce Screen (Italian Republic Law 130/2023)**	Italy	Capillary blood AAB testing	Children aged 1–17 years	Registry enrollment, symptom education, and specialist referral	Expected reduction in DKA through early-stage identification and intervention	[57,58,59]
**TRIGR**	International	High-risk infant cohort surveillance	Children with genetic susceptibility	Longitudinal follow-up	Exhibited value of structured monitoring and early detection	[30]
**INNODIA DETECT**	International	Islet AABs	Relatives and the general population	Ongoing monitoring of at-risk individuals	Reduced incidence of severe DKA at onset to below 5%	[60]
**Fr1da**	Bavaria, Germany	Islet AAB screening	Children aged 2–5 years	Metabolic surveillance, family education, and specialist follow-up	DKA at diagnosis was approximately 3.2%, substantially lower than unscreened populations	[45]
**GPPAD**	International	Genetic risk screening followed by serial islet AAB monitoring	Genetically at-risk newborns and infants (under 5 months old)	Longitudinal surveillance and prevention trial enrollment	Early identification of presymptomatic T1D; DKA outcome data remain limited	[61]
**ASK**	USA	Islet AAB screening	Children aged 1–17 years	Risk stratification, education, and longitudinal follow-up	Early identification of presymptomatic T1D with low DKA rates among monitored children	[62]

TEDDY—The Environmental Determinants of Diabetes in the Young; USA—the United States; AAB—autoantibody; DKA—diabetic ketoacidosis; T1D—type 1 diabetes; TRIGR—Trial to Investigate Glucose Tolerance; GPPAD—Global Platform for the Prevention of Autoimmune Diabetes; D1Ce—diabetes type 1 and celiac; ASK—Autoimmunity Screening for Kids.

**Table 2:** Overview of pharmacologic advances in type 1 diabetes prevention and disease.

Therapy	Mechanism	Study/Trial	Population	Key Outcome	Regulatory Status	Ref.
**Teplizumab**	Humanized anti-CD3 monoclonal antibody	TN-10 study	Stage 2 T1D	Delay of progression from stage 2 to stage 3 by about 2 years	FDA-approved; recent EMA approval	[47,50,51,68,70,71]
**Regulatory T-cell (Treg) therapy**	Adoptive transfer of Tregs	Randomized trial	Pediatric new-onset T1D	Safe but with limited efficacy	Investigational	[72]
**Oral insulin**	Antigen-specific immune tolerance	DPT-1, TrialNet, POInT	Autoantibody-positive individuals	Delayed progression in a specific subgroup; no overall benefit	Investigational	[73,74,75,76,77,78]
**Low-dose IL-2**	Expansion of Tregs	Early-phase trials	Early T1D	Safe; increases Treg numbers	Investigational	[79,80,81]
**Anti-IL-21 monoclonal antibodies**	Expansion and activation Tregs	Clinical trials	Adults with stage 3 T1D	Preservation of β-cell function	Investigational	[64,82,83,84]
**JAK inhibitors (e.g., baricitinib, abrocitinib, ritlecitinib)**	Disruption of JAK-STAT signaling pathway	Clinical trials	Stage 3 T1D	Preservation of residual β-cell function and improvement of glycemic control	Investigational	[64,82,83,84]
**Verapamil**	β-cell protection via TXNIP suppression	Randomized clinical trials	Newly diagnosed pediatric T1D	Modest preservation of C-peptide secretion	Investigational adjunct	[85,86]
**Immunotherapies**						
**Abatacept**	Selection modulation of T-cell co-stimulation	Clinical trials	Early-stage or recent-onset T1D	Temporary β-cell preservation	Investigational	[66,69]
**Rituximab**	CD20+ β-cell depletion	Clinical trials	Various disease stages	Temporary preservation of C-peptide	Investigational	[66,69]
**Anti-thymocyte globulin (ATG)**	Broad depletion and modulation of T-lymphocytes	Clinical trials	Recent-onset or presymptomatic T1D	Slowing the decline in endogenous insulin production	Investigational	[66,69]
**Golimumab**	Inhibition of tumor necrosis factor-alpha (TNF-α)	Clinical trials	New-onset/newly diagnosed T1D (stage 3) or pre-symptomatic T1D (stage 2)	Preservation of β-cell function	Investigational	[20,87]

T1D—type 1 diabetes; FDA—Food and Drug Administration; EMA—European Medicines Agency; Treg—regulatory T-cell; DPT-1—Diabetes Prevention Trial of Type 1, POInT—Primary Oral Insulin Trial; IL-2—interleukin-2; Anti-IL-21—anti-interleukin-21; JAK inhibitor—Janus kinase inhibitor; JAK-STAT—Janus kinase and signal transducer and activator of transcription; ATG—anti-thymocyte globulin; TNF-α—tumor necrosis factor-alpha.

## Data Availability

No new data were created or analyzed in this study. Data sharing is not applicable to this article.

## Abbreviations

The following abbreviations are used in this manuscript:

DKA	Diabetic ketoacidosis
T1D	Type 1 diabetes
HDI	Human development index
COVID-19	Coronavirus 2019
SARS-CoV-2	Severe acute respiratory syndrome coronavirus 2
AKI	Acute kidney injury
TEDDY	The Environmental Determinants of Diabetes in the Young
TRIGR	Trial to Investigate Glucose Tolerance
ISPAD	International Society for Pediatric and Adolescent Diabetes
AAB	Autoantibody
JDRF	Juvenile Diabetes Research Foundation
ADA	American Diabetes Association
IGT	Impaired glucose tolerance
OGTT	Oral glucose tolerance test
HbA1c	Glycosylated hemoglobin
HR	Hazard ratio
CI	Confidence interval
GADA	Glutamic acid decarboxylase autoantibody
IAA	Insulin autoantibody
IA-2A	Insulinoma-associated antigen-2 autoantibody
ICA	Islet cell autoantibody
ZnT8A	Zinc transporter 8 autoantibody
FDA	U.S. Food and Drug Administration
Treg	Regulatory T-cell
IL	Interleukin
MHRA	Medicines and Healthcare products Regulatory Agency
TCR	T cell receptor
CAR	Chimeric antigen receptor
MDSC	Myeloid-derived suppressor cell
MSC	Mesenchymal stem cell
GALT	Gut-associated lymphoid tissue
DPT-1	Diabetes Prevention Trial of Type 1
FPIR	First-phase insulin release
Anti-IL	Anti-interleukin
JAK	Janus kinase
ER	Endoplasmic reticulum
ATG	Anti-thymocyte globulin
